# Knowledge, Attitudes, and Practices of Hand Hygiene, Mask Use, and Social Distancing among Public Hospital and Polyclinic Nurses in Barbados during the Coronavirus 2019 Pandemic

**DOI:** 10.3390/epidemiologia5010008

**Published:** 2024-03-06

**Authors:** Uma Gaur, Wendy Sealy, Ambadasu Bharatha, Natasha P. Sobers, Kandamaran Krishnamurthy, Michael H. Campbell, Cara Cumberbatch, Maia Drakes, Marielle Gibbs, Charisse Alexander, Heather Harewood, O. Peter Adams, Subir Gupta, Ali Davod Parsa, Russell Kabir, Md Anwarul Azim Majumder

**Affiliations:** 1 Faculty of Medical Sciences, The University of the West Indies, Cave Hill Campus, Bridgetown BB11000, Barbados; uma.gaur@cavehill.uwi.edu (U.G.); wendy.sealy@cavehill.uwi.edu (W.S.); ambadasu.bharatha@cavehill.uwi.edu (A.B.); kandamaran.krishnamurthy@cavehill.uwi.edu (K.K.); michael.campbell@cavehill.uwi.edu (M.H.C.); crc.cara@gmail.com (C.C.); maia.drakes@gmail.com (M.D.); mariellegibbs@gmail.com (M.G.); c.risse99@hotmail.com (C.A.); heather.harewood@cavehill.uwi.edu (H.H.); peter.adams@cavehill.uwi.edu (O.P.A.); subir.gupta@cavehill.uwi.edu (S.G.); 2 George Alleyne Chronic Disease Research Centre, The University of the West Indies, Cave Hill Campus, Bridgetown BB11000, Barbados; natasha.sobers@cavehill.uwi.edu; 3 School of Allied Health and Social Care, Faculty of Health, Medicine and Social Care, Anglia Ruskin University, Chelmsford CM1 1SQ, UK; ali.parsa@aru.ac.uk (A.D.P.); russell.kabir@aru.ac.uk (R.K.)

**Keywords:** COVID-19 pandemic, nurses, hand washing, mask wearing, social distancing

## Abstract

Background: Nurses are essential members of the healthcare workforce and were among the first-line carers for patients in community and hospital settings during the COVID-19 pandemic. As a result, they were at a heightened risk of infection, resulting in several reported deaths among nursing staff. Several preventive measures were adopted to contain the spread of the COVID-19 virus. This study aims to explore the knowledge, attitudes, and practices (KAP) of nurses regarding hand hygiene, mask wearing, and social distancing measures in healthcare settings in Barbados during the COVID-19 pandemic. Method: An online survey of nurses working in public hospitals and polyclinics (public primary care clinics) in Barbados from March 2021 to December 2021 was conducted. A nonsystematic convenience sampling method was employed to recruit nurses who were readily available and willing to participate. A questionnaire captured the sociodemographic information and knowledge and practices related to hand hygiene, the use of face masks, and social distancing. Each correct response received one mark. Overall knowledge scores were categorized as poor (<60%), average (60–80%), or good (>80–100%). Results: Of the 192 participants, the majority were female (82.8%) and had >5 years of experience (82%). The findings revealed that 45.8% had poor knowledge of hand hygiene, and that the knowledge of 43.8% of respondents was average. Multivariable logistic regression showed that, after adjustment for age and gender, registered nurses had 2.1 times increased odds (95% confidence interval 1.0, 4.2) of having good knowledge compared to other nursing categories. Regarding mask wearing, 53.6% of nurses had average knowledge, and 27.1% had good knowledge. Multivariable logistic regression showed that, after adjustment for age and gender, registered nurses had 3.3 times increased odds (95% confidence interval 1.5, 7.4) of having good knowledge compared to nursing assistants. A total of 68.6% of respondents followed the correct steps of handwashing every time, and 98.3% wore a mask in public places. More than half of the nurses (51.2%) kept a safe distance from others to avoid spreading SARS-CoV-2; one-third were in a crowded place(s) in the past three months, and 55.8% usually followed guidelines for social isolation as recommended by the WHO. Conclusions: The study identified knowledge deficiencies related to hand hygiene and wearing masks among nurses. It is imperative to provide additional training on infection control measures.

## 1. Introduction

The coronavirus 2019 (COVID-19) pandemic has caused challenges for researchers and healthcare professionals worldwide. As of April, 2023, the COVID-19 pandemic has caused more than 685 million infections and 6.8 million deaths [1]. Globally, an estimated 80,000 to 180,000 healthcare workers (HCWs) died from COVID-19 between January 2020 and May 2021 [2]. Vaccines against SARS-CoV-2 have been developed at an unprecedented pace. While vaccines have been shown to prevent illness effectively, their ability to effectively prevent transmission is less clear [3]. Fully vaccinated individuals have the potential to transmit the virus without exhibiting symptoms, and effective and practical preventive measures to reduce the risk of community transmission are needed. Further, the knowledge, attitudes, and practices (KAP) of healthcare professionals, particularly nurses, who have frequent close contact with patients, regarding infection control procedures are a key component of these efforts [4].

The World Health Organization (WHO) has recommended several hygiene measures and behavioral guidelines to prevent COVID-19 [5,6], including wearing masks, maintaining safe physical distance, and sanitizing hands. The uptake of these necessary cost-effective, evidence-based, nonpharmaceutical interventions (NPIs) to minimize the transmission of infection and associated mortality is predicated upon community buy-in [7]. Thus, assessment of KAP is essential for implementing behavioral change [8] and provides insight for addressing knowledge gaps and misconceptions regarding the transmission of infection [9].

The first case of COVID-19 in Barbados was reported on 17 March 2020 [10]; subsequently, the country adopted multiple strategies to combat the epidemic, including the rapid and broad deployment of vaccines [11]. However, vaccination rates plateaued at about 57%, and vaccine hesitancy has been a barrier to further uptake [12]. Importantly, vaccines do not eliminate the need for NPIs. Precautions, such as hand hygiene, mask use, and social isolation, remain essential to reducing transmission [10].

Given the pivotal role of nurses (registered nurses, nursing assistants, and midwives) in infection control, understanding their KAP regarding NPIs in varied practice contexts is essential [13]. This study, therefore, aimed to assess the KAP of nurses regarding hand hygiene, mask use, and social distancing measures in healthcare settings in Barbados during the pandemic. These findings may contribute to developing targeted interventions to improve adherence to NPIs among nurses, thereby enhancing the effectiveness of infection control in Barbados and other small island developing states (SIDS).

## 2. Material and Methods

### 2.1. Study Design

A cross-sectional study of nurses (registered nurses, nursing assistants, and midwives) employed at selected hospitals and public primary care clinics in Barbados was conducted during the COVID-19 pandemic. The data were gathered via an online REDCap survey link distributed from March 2021 to December 2021. Research Electronic Data Capture (REDCap) is a secure, web-based application designed specifically for data collection and management in research studies.

### 2.2. Study Setting

Participants included registered nurses, nursing assistants, and midwives from the following public healthcare facilities in Barbados: (a) Queen Elizabeth Hospital (QEH) (lone tertiary hospital), (b) Geriatric Hospital (St. Michael District Hospital—main residential geriatric facility), (c) Psychiatric Hospital, and (d) Branford Taitt Polyclinic (public primary care clinic—second largest in terms of population served). Barbados is a SIDS nation in the southern Caribbean and experiences the associated vulnerabilities of economic and other resource limitations [14].

### 2.3. Recruitment of Study Participants

A nonsystematic convenience sampling method was employed to recruit nurses who were willing to participate. All nurses (registered nurses, nursing assistants, and midwives) working at the abovementioned healthcare facilities were eligible for inclusion in the study. The hospital administration provided email addresses for the nursing staff. Those who consented to participate were enrolled sequentially. Nurses received an invitation to participate along with the REDCap survey link via email.

### 2.4. Study Instruments

The study utilized a pretested, self-administered questionnaire [15,16,17,18,19,20] consisting of the following sections:

Demographics: Demographic details included age, gender, marital status, hospital, job characteristics, formal training, and education level.

A. Hand Hygiene:

i. Knowledge of hand hygiene: The WHO Hand Hygiene Knowledge Questionnaire [15] was employed to evaluate (1) hygiene knowledge training, main routes of cross-transmission, the most frequent source of germs responsible for infections, and the prevention of germ transmission and (2) the minimal time needed for hand rub, as well as other hand hygiene methods to avoid harmful colonization of pathogens on the hands.

Ten questions were used to assess hand hygiene knowledge. The first question queried training during the last three years, and the second question queried compliance with the routine use of an alcohol-based sanitizer. The remaining eight questions, collectively comprising 25 items, assessed the level of knowledge on hand hygiene. For each correct response, participants scored one point. Participants’ scores were totaled, and the overall knowledge scores were categorized as poor (<60%), moderate (60–80%), or good (80–100%) [16].

ii. Hand hygiene practice and attitudes: The researchers devised four questions to assess individuals’ handwashing practices and attitudes. These questions were crafted following a thorough review of pertinent literature [19,20].

B. Mask wearing:

Knowledge and practice of mask wearing: A questionnaire developed by Kumar et al. [17] was used to assess knowledge and practice of mask wearing. Each correct answer scored one (1) and each incorrect answer scored zero (0). The total number of questions was nine, and the final score was calculated and then labelled according to the percentage of correct responses as good (>80%), moderate (60–80%), and poor (<60%).

C. Social Distancing:

Practice of social distancing: This section consisted of seven questions. Five questions were taken from a questionnaire used by Al-Hanawi et al. [18]. The researchers added two questions to elicit additional information on social distancing practices.

### 2.5. Statistical Analysis

The Stata Statistical Package version 17 was used to analyze the data. Descriptive statistics were used to summarize demographic variables. For the knowledge, perception, and practice items, means and standard deviations were reported. Shapiro–Wilk W and Shapiro-Francia tests for normality were performed. Variables found to be not normally distributed were given as median and interquartile range (IQR). Inferential statistics were performed to examine the predictors of hand hygiene knowledge and mask wearing using bivariate analysis. We examined the predictors of both hand hygiene knowledge and mask wearing knowledge using bivariate analysis. The independent predictors examined were age, gender (males versus females), educational level (bachelor’s degree or higher versus associate degree/certificate), and category of nurse (registered nurse or not). Given that the category of nurse was the only statistically significant independent risk factor after bivariate analysis, only age and gender were entered into the final multivariable model to adjust for these as potential confounders. We also explored the possible associations of the other predictors in models using backward selection (*p* > 0.1), but none of the other predictors were statistically significant at *p* < 0.1.

### 2.6. Ethical Approval

The study protocol was approved by The University of the West Indies, Barbados Ministry of Health Research Ethics Committee/Institutional Review Board (IRB) (IRB no: 210202). An information sheet was presented to the participants online before the survey instrument. Participants indicated their consent by proceeding with the survey. The study adhered to the principles of the Declaration of Helsinki.

## 3. Results

### 3.1. Demographics of the Respondents

Table 1 presents the demographic characteristics of the respondents. Of the 285 individuals invited, 192 completed the questionnaire, resulting in a response rate of 67.4%, which formed the basis of our analysis. The median age of the respondents was 37.5 years (IQR 32.47). Females comprised the majority (82.8%) of the participants. In terms of qualifications, 44.8% of the respondents possessed graduate-level qualifications, and 44.8% held associate degrees/certificates. Additionally, 157 (81.8%) had accumulated five or more years of experience, while 35 (18.2%) had less than five years of experience. Most participants were registered nurses (76.6%), followed by assistant nurses (18.2%) and midwives (12.0%).

### 3.2. Findings of Hand Hygiene

#### 3.2.1. Knowledge of Hand Hygiene

The findings indicated that 45.8% of respondents had poor knowledge of hand hygiene, 43.8% had moderate knowledge, and 10.4% had good knowledge. Approximately 62% of participants demonstrated knowledge of primary routes of cross-transmission of germs between patients when their hands were not clean. However, approximately 35% of respondents indicated that germs already present on or within the patient are the primary source of healthcare-associated infections. On the other hand, 93.8% of respondents acknowledged that practicing hand hygiene prevented the transmission of germs before encountering a patient. Further, majorities, 86.5% and 63% respectively, acknowledged the importance of avoiding artificial fingernails and damaged skin due to their association with an increased risk of harmful germ colonization on the hands (Table 2).

#### 3.2.2. Practices and Attitude of Hand Hygiene

Table 3 summarizes participant responses regarding hand hygiene practices in clinical settings. The majority (85.9%) reported adhering to hand hygiene practices by washing their hands before touching a patient, between caring for individual patients, and immediately after completing a clean aseptic procedure. Hand washing with soap and water emerged as the preferred hand hygiene procedure among the participants, while more than 90% of the participants reported washing their hands more than 10 times in the clinical setting. However, only 69% of the participants reported completing all the correct handwashing steps every time, and just about one-third of the participants reported washing their hands after touching a clean surface.

#### 3.2.3. Predictors of Hand Hygiene Knowledge

Bivariate analysis showed that only the category of nurse was a significant predictor of hand hygiene knowledge (Table 4). When the category of nurse was entered into a multivariable logistic regression, analysis showed that after adjustment for age and gender, registered nurses had 2.1 times increased odds (95% CI 1.0, 4.2) of having good knowledge compared to other nursing categories (nursing assistants/midwives).

### 3.3. Findings for Mask Wearing

#### 3.3.1. Knowledge of Mask Wearing

Regarding wearing masks, 53.6% of respondents had moderate knowledge, 27.1% had good knowledge, and 19.3% had poor knowledge. Additionally, more than 95% knew the correct way of wearing a surgical mask, 79.2% also knew that there are three layers in a surgical mask, and 80.1% knew how to identify the correct filter media barrier. Moreover, 88.3% of participants knew that surgical masks were effective against COVID-19, while only 6.3% were aware of the maximum duration of wearing a face mask. When asked about the extent to which a surgical mask should cover the face, 92.0% answered correctly, and all the respondents correctly reported the purpose of the metal strip (Table 5).

#### 3.3.2. Practice and Attitude of Mask Wearing

The majority of the participants reported removing their masks when there is a need to talk to the patient during clinic time (98.3%), 97.7% do not store the used surgical mask in a bag for later use, and wearing a mask in public places to protect themselves against COVID-19 was indicated by 98.3% of the respondents. Further, 82.0% of persons identified red-coded bags for disposing masks. Black-coded bags were the second most reported choice and was indicated by 14.5% of the participants (Table 6).

#### 3.3.3. Predictors of Mask Wearing Knowledge

Bivariate analysis revealed that only the category of nursing staff predicted mask wearing knowledge (Table 7). When this variable was entered into a multivariable logistic regression it showed that after adjustment for age and gender, registered nurses had 3.3 times increased odds (95% CI 1.5, 7.4) of having good knowledge compared to other nursing categories.

### 3.4. Findings of Social Distancing

#### Practice of Social Distancing

Nearly all participants (98.8%) reported actively practicing social distancing. Many participants reported avoiding cultural behaviors, such as shaking hands (92.7%) and washing their hands with soap and water for at least 40 s, especially after going to a public place or after sneezing, coughing, or blowing their nose (90.8%). A minority of participants (12.2%) disclosed their involvement with more than 20 people or visiting crowded places. However, a significant majority of participants reported adhering closely to social isolation guidelines, with 55.8% mostly following the rules and 43.6% strictly abiding by them (Table 8).

## 4. Discussion

This study examines KAP among public hospital and polyclinic nurses in Barbados regarding hand hygiene, mask wearing, and social distancing. Findings can inform targeted interventions, educational campaigns, and training programs to promote adherence to infection control measures by nursing staff, thereby enhancing personal protection and overall control of the pandemic.

Although based in Barbados, our study may have broader implications by offering findings that can inform infection control efforts in other regions, especially SIDS and other countries with similar resource-limited healthcare contexts. Our study is particularly relevant given the pivotal role of nurses in both infection control and patient education on NPIs. Our research further provides valuable information on the preparedness and response of frontline HCWs, highlighting the strengths and gaps in current public health strategies. These insights may be useful for other countries facing comparable healthcare challenges, thereby contributing to the global understanding of the implementation of infection control measures in pandemic scenarios.

### 4.1. Hand Hygiene

Most nurses in our study believed that cross-transmission of potentially harmful germs occurs due to poor hand hygiene. However, 45.8% of respondents had poor knowledge of hand hygiene. Enhancing hand hygiene compliance among HCWs significantly reduces spread of the COVID-19 infections. Previous studies have shown that hand hygiene is the most effective way to prevent the spread of COVID-19 infection, taken together with other protective measures such as wearing masks and practicing social distancing [5,6].

In the current study, most participants knew that jewelry and rings should be removed before handwashing (86.5%) and that artificial fingernails are a common source of germs (90.1%). Indeed, longstanding guidelines established in the US by Siaman et al. [21] prohibit use of artificial nails. Further, 89.1% respondents believed that using proper hand hygiene technique after patient contact can prevent infection transmission, and 89.5% washed their hands before touching a patient. Hand hygiene knowledge and practice in Barbados compare favorably to findings in diverse settings globally, including Iran, where 66.2% of nurses agreed that hands should be washed before any procedure or patient contact [22]; Norway, where hand hygiene adherence among nurses and nursing students was 58% [23]; and Tanzania, where a large-scale study found inadequate hand hygiene in inpatient and outpatient clinical settings [24]. Nurses’ knowledge and practice of hand hygiene is especially important, because they are role models for good infection control practice [25]. Although the effectiveness of hand hygiene in reducing SARS-CoV-2 transmission is not without question [26], its role as a preventive measure is broadly important, given that similar viruses (e.g., SARS-CoV, influenza and MERS-CoV) can survive on surfaces for a long time [27].

Knowledge regarding alcohol-based hand sanitizing among nurses in our study was varied. Only 39.1% of participants correctly indicated that hand rubbing was a faster method of hand sanitizing than handwashing, and 66.1% believed that handwashing and hand rubbing should be performed in sequence, which is incorrect. An encouraging finding was that the vast majority of nursing staff (93.2%) correctly indicated that hand washing is more effective than sanitizing with alcohol. These findings underscore the need for nurses to access appropriate training in hand hygiene. CDC recommendations specify using an alcohol-based hand rub that contains 60% alcohol if soap and water are not available [28]. However, correct technique is important to ensure the effectiveness of alcohol-based hand rubs.

### 4.2. Mask Wearing

The WHO strongly advocates use of face masks when interacting with patients to prevent infection transmission [29,30]. Face masks are an essential component of personal protective equipment (PPE), and correct usage is crucial to achieve optimal protection against infections [31]. Masks are especially important for nursing staff, because social distancing and remote work are usually not possible for personnel providing hands-on care [32]. Consequently, the provision of PPE to nursing staff is a priority [10,32], including gloves, eyewear, surgical face masks (SFM), and filtering face piece (FFP) masks to prevent viral spread through contact or droplet transmission.

The majority of participants (88.3%) understood that wearing masks can effectively safeguard against COVID-19 infection, and 95.4% demonstrated awareness of the correct method for wearing masks. These findings compare favorably to a recent study in Pakistan (70.9% and 43.6%, respectively) (16). However, an Egyptian study reported that a minority of nurses (19.2%) indicated that mask wearing effectively reduces risk COVID-19 infection [33].

Our study found that 88.9% of participants considered cloth masks to be ineffective. This finding is consistent with empirical studies [34,35,36] demonstrating that cloth masks are one-third less effective than medical masks. As research findings suggest that cloth masks play a limited role in reducing the risk of COVID-19 virus exposure, these findings underscore the importance of training regarding differential effectiveness and appropriate face mask use [37].

In this study, 98.3% of participants reported wearing a mask in public to protect against COVID-19. Public mask use in Barbados was higher than among Saudi Arabian HCWs (93.8%) and Canadian nurses (89%) [38,39]. Numerous studies have demonstrated a strong correlation between mask use and declines in COVID-19 incidence [40,41,42,43,44].

A key finding of our study was that 98.3% of nursing staff removed their masks while speaking, signaling a significant discrepancy between knowledge and practice. This emphasizes the importance of integrating practical application with theoretical knowledge in nursing education, particularly in high-risk health settings [13]. Our findings may inform future training and public health interventions in Barbados and more broadly to promote translation of mask use knowledge to practice for COVID-19 prevention [45].

Multivariate analysis demonstrated that registered nurses have better knowledge of hand hygiene and mask wearing compared to other nursing categories with less advanced training. The differential knowledge gap indicates a need for targeted training and policy development across all levels of nursing staff [46]. These knowledge disparities are not limited to Caribbean settings and have relevance for guiding global health strategies [47]. The important roles of nurses in modeling best practice for infection control necessitates enhanced training and policy frameworks [48].

### 4.3. Social Distancing

In this study, 98.8% of nursing staff reported adhering to social distancing guidelines generally, but only 51.2% indicated that they maintained a safe distance all the time. In comparison, a US general population study [49] found that 31.3% followed social distancing guidelines. Similarly, a study in Egypt found that 40% of nurses usually practiced social distancing during the pandemic [50]. Adherence to isolation guidelines was less robust, with 43.6% of nurses in Barbados indicating that they strictly followed rules for social isolation. Findings regarding social distancing and isolation should be interpreted considering that nurses, as frontline HCWs, were exempt from isolation rules that would have impeded clinical duties requiring physical proximity. In contrast, 92.7% of respondents indicated that they avoided cultural behaviors associated with risk of infection, such as shaking hands These findings are consistent with a study of HCWs in Egypt, in which respondents were highly adherent to most NPIs with the exception of social distancing. An important context is that both the Barbadian and Egyptian studies were conducted during the height of the pandemic, when government-instituted guidelines for social distancing were most strict and public awareness campaigns were most active. Vigorous public awareness campaigns were likely to have increased knowledge among HCWs and the general population. Given the constraint of necessary close contact between patients and nurses and, hence, the limited applicability of social distancing, the implication is that nurses need to strictly adhere to the other NPIs and correctly and consistently use PPE to reduce transmission risk.

However, there are significant organizational challenges in providing adequate PPE [10]. These challenges include logistical difficulties in PPE supply and distribution, and the increased burden on healthcare systems during peak pandemic periods [51]. These barriers can compromise safety protocols and increase risk of infection for HCWs and patients [52].

### 4.4. Limitations of the Study

This study is subject to several limitations, including a relatively small sample size (n – 192), incomplete questionnaire responses, and low response rate, which was probably exacerbated by online data collection. There is also potential for selection bias due to convenience sampling. Response bias may also be present, as self-reported answers could be influenced by social desirability, particularly in a cultural context where certain health practices are highly valued.

## 5. Conclusions

Findings for Barbados indicated that nurses showed good knowledge of hand hygiene and correctly identified the primary route of cross-transmission of harmful germs between patients. However, certain knowledge gaps were observed, particularly concerning the utilization of alcohol-based hand rubs. The majority of nurses identified the appropriate method for masking to protect against COVID-19. Nonetheless, there were some knowledge gaps concerning the types of masks that offer adequate protection against COVID-19 and the recommended duration for mask wearing. This study underscores the need for continuous education and training to enhance the knowledge and adherence to hand hygiene and mask wearing practices among HCWs, particularly in resource-limited settings.

## Figures and Tables

**Table 1:** Demographic characteristics of respondents.

Variable	Frequency	%
Age (years) (n = 180)		
15–25	8	4.2
26–35	67	34.9
36–45	54	28.1
46–55	28	14.6
≥55	23	11.9
Gender (n = 192)		
Females	159	82.8
Males	33	17.2
Education (n = 189)		
Ph.D.	2	1.0
Master’s degree	15	7.8
Associate degree	76	39.5
Bachelor’s degree	66	34.4
Certificate	30	15.6
Profession * (n = 184)		
Registered nurse	147	76.6
Midwife	23	12.0
Nursing assistant	35	18.2
Department (n = 188)		
ICU	17	8.9
Paediatrics	11	5.7
Internal Medicine	6	3.1
Geriatric	11	5.7
Surgery	26	13.5
Obstetrics	13	6.8
Psychiatry	46	24.0
Medicine	21	11.0
Emergency	4	2.1
Outpatient Clinic	8	4.2
Other	25	13.0
Formal hand hygiene training in the last three (3) years (n = 187)		
Yes	152	79.2
No	35	18.2

* The categories of profession are not mutually exclusive (e.g., 20 registered nurses are also midwives).

**Table 2:** Knowledge of hand hygiene in nurses, nursing assistants, and midwives (N-192).

Knowledge Items	Frequency	Percent
**Which of the following is the main route of cross-transmission of potentially harmful germs between patients in a healthcare facility?**		
Healthcare workers’ hands when not clean (Yes)	114	62.0
Air circulating in the hospital (No)	2	1.1
Patients’ exposure to colonized surfaces (i.e., beds, chairs, tables, floors) (No)	28	15.2
Sharing noninvasive objects (i.e., stethoscopes, pressure cuffs, etc.) between patients (No)	40	21.7
2. **What** **is the most frequent source of harmful pathogens responsible for healthcare-associated infections?**		
The water system (No)	9	4.7
Pathogens already present on or within the patient (Yes)	67	34.9
The ventilation system within the hospital (No)	7	3.6
The hospital environment (No)	109	56.8
3. **Which of the following hand hygiene actions prevents transmission of germs to the patient?**		
Before touching a patient (Yes)	180	93.8
Immediately after the risk of body fluid exposure (No)	108	56.2
After exposure to the immediate surroundings of a patient (No)	101	52.6
Immediately before a clean/aseptic procedure (Yes)	127	66.2
4. **Which of the following hand hygiene actions prevents transmission of germs to the healthcare worker?**		
After touching a patient (Yes)	171	89.1
Immediately after the risk of body fluid exposure (Yes)	136	70.8
Immediately before a clean/aseptic procedure (No)	128	66.7
After exposure to the immediate surroundings of a patient (Yes)	89	46.4
5. **Which of the following statements on alcohol-based hand rub and handwashing with soap and water are true?**		
Hand rubbing is more rapid for hand cleansing than handwashing (True)	75	39.1
Hand rubbing causes skin dryness more than handwashing (False)	60	31.2
Hand rubbing is more effective against germs than handwashing (True)	13	6.8
Handwashing and hand rubbing are recommended to be performed in sequence (False)	127	66.2
The minimal time needed for an alcohol-based hand rub to kill most germs on your hands (20 s)	77	40.1
6. **Which of the following should be avoided, as associated with increased likelihood of colonization of hands with** **harmful** **germs?**		
Wearing jewelry (Yes)	166	86.5
Damaged skin (Yes)	121	63.0
Artificial fingernails (Yes)	173	90.1
Regular use of a hand cream (No)	27	14.1

**Table 3:** Practice and attitude of hand hygiene (N-192).

Practice Items	Frequency	Percent
**In which of the following clinical situations do you wash your hands?**		
Before touching a patient	165	85.9
Between caring for individual patients	165	85.9
Immediately before commencing a clean aseptic procedure	175	91.2
Immediately after completing a clean aseptic procedure	165	85.9
2. **In which of the following clinical situations do you wash your hands?**		
After touching a clean surface in the clinical area	63	32.8
Other	27	14.1
3. **How often do you wash your hands while in the clinical setting?**		
>10 times	171	91.4
6–9 times	12	6.4
3–5 times	4	2.1
4. **Do you complete all of the correct steps of the handwashing process every time?**	**129**	**68.6**
5. **Which of the following procedures do you prefer to use in the clinical setting?**		
Hand washing with soap and water	182	97.3
Rubbing with alcohol	5	2.7

**Table 4:** Predictors of hand hygiene knowledge.

Characteristics	Moderate/Good Knowledge, n (%)	OR, *p*-Value
Male, n = 33	16 (48.5%)	0.76, *p* = 0.472
* Female, n = 159	88 (55.4%)	
Bachelor’s degree or higher, n = 86	51 (59.3%)	1.46, *p* = 0.199
* Associate degree/Certificate, n = 106	53 (50.0%)	
Greater than or equal to 5 years’ experience, n = 157	85 (54.1%)	0.99, *p* = 0.988
* Less than 5 years’ experience, n = 35	19 (54.3%)	
Registered nurse, n = 147	86 (58.5%)	2.11, *p* = 0.031
* Nursing assistant, n = 45	18 (40.0%)	

* Denotes this group is the reference group in the bivariate analysis.

**Table 5:** Knowledge of mask wearing.

Knowledge Items	Correct Answer	
	Frequency	Percent
**Which is the correct way of using a surgical face mask to protect against COVID-19**		
White side facing out	8	4.6
White side facing in (Correct)	166	95.4
**How many layers are there in a surgical mask?**		
Two	33	19.1
Three (Correct)	137	79.2
Four	3	1.7
**Can wearing a surgical mask protect you from COVID-19?**		
Yes (Correct)	151	88.3
No	20	11.7
**Which layer acts as a filter media barrier?**		
First layer	29	17.0
Middle layer (Correct)	137	80.1
Last layer	5	2.9
**Which type of masks actually protect against COVID-19?**		
97% BFE and PFE	54	34.8
95% BFE and PFE (Correct)	99	63.9
91% BFE and PFE	2	1.3
**How long can you wear a surgical mask?**		
1 h	4	2.3
2 h	16	9.2
4 h	143	82.2
8 h (Correct)	11	6.3
**For proper wearing, to what extent should the surgical mask cover the face?**		
Nose only	0	0
Nose and mouth	14	8.0
Nose, mouth and chin (Correct)	160	92.0
**What is the purpose of the metal strip on a surgical mask?**		
To fit on the nose (Correct)	173	100
To fit on the chin	0	0
**Is the cloth facial mask as effective as a regular surgical facial mask?**		
Yes	19	11.1
No (Correct)	152	88.9

**Table 6:** Practices and attitudes regarding mask wearing (N-173).

Practice Items	Frequency	Percent
**During clinics, if there is a need to talk to the patient, do you remove your mask?**		
Yes	170	98.3
No	3	1.7
**If you are not sick, do you store the used surgical mask in a bag for later use?**		
Yes	4	2.3
No	169	97.7
**Do you wear a mask in public places to protect yourself against COVID-19?**		
Yes	170	98.3
No	3	1.7
**In which color-coded bag do you dispose of your mask?**		
Red-coded bag	141	82.0
Yellow-coded bag	4	2.3
Black-coded bag	25	14.5
Blue-coded bag	2	1.2

**Table 7:** Predictors of mask wearing knowledge.

Characteristics	Moderate/Good Mask Wearing Knowledge, n (%)	OR, *p*-Value
Male, n = 33	28 (84.8)	1.41, *p* = 0.511
* Female, n = 159	127 (79.9)	
Bachelor’s degree or higher, n = 86	71 (82.6)	1.24, *p* = 0.563
* Associate degree/Certificate, n = 106	84 (79.2)	
Greater than 5 years’ experience, n = 157	125 (79.6)	0.65, *p* = 0.411
* Less than 5 years’ experience, n = 35	30 (85.7)	
Registered nurse, n = 147	126 (85.7)	3.31, *p* = 0.002
* Nursing assistant, n = 45	29 (64.4)	

* Denotes the reference group in the analysis.

**Table 8:** Practice of social distancing.

Social Distancing Items	Frequency	Percent
**Do you keep your distance from others to avoid spreading SARS-CoV-2? (N = 164)**		
Yes all	84	51.2
Yes sometimes	77	47.0
No	3	1.8
2. **Have you, in the past 3 months, been to a social event involving more than 20 people? (N = 164)**		
Yes	20	12.2
No	144	87.8
3. **Have you, in the past 3 months, been to a crowded place? (N = 162)**		
Yes	54	33.3
No	108	66.7
4. **Have you, in the past 3 months, avoided cultural behaviors, such as shaking hands? (N = 164)**		
Yes	152	92.7
No	12	7.3
5. **Have you been practicing social distancing? (N = 164)**		
Yes	162	98.8
No	2	1.2
6. **Recently, have you washed your hands with soap and water, for at least 40 s, especially after going to a public place, or after blowing, coughing, or sneezing? (N = 163)**		
Yes	148	90.8
No	15	9.2
7. **Do you closely follow social isolation rules? (N = 163)**		
Yes mostly	91	55.8
Yes strictly	71	43.6
No	1	0.6

## Data Availability

N.P.S., M.C.H., and M.A.A.M. have full access to all data. This data is not publicly accessible but are available from the corresponding author upon reasonable request.
